# Parent Prediction of Child Mood and Emotional Resilience: The Role of Parental Responsiveness and Psychological Control

**DOI:** 10.1155/2011/375398

**Published:** 2011-11-03

**Authors:** Kristy L. Boughton, Margaret N. Lumley

**Affiliations:** Clinical Psychology: Applied Developmental Emphasis, Department of Psychology, University of Guelph, Guelph, ON, Canada N1G 2W1

## Abstract

Research consistently shows low to moderate agreement between parent and child reports of child mood, suggesting that parents are not always the best predictors of child emotional functioning. This study examines parental responsiveness and psychological control for improving prediction of early adolescent mood and emotional resilience beyond parent report of child emotional functioning. Participants were 268 early adolescents administered measures of depression symptoms, emotional resilience, and perceptions of parenting. Parents of participating youth completed measures of youth emotional functioning. Parental responsiveness and psychological control each emerged as family variables that may be of value for predicting child emotional functioning beyond parent reports. Specifically, responsiveness explained significant variance in child depression and resilience after accounting for parent reports, while parental psychological control increased prediction of child mood alone. Results generally suggest that parenting behaviours may be an important consideration when children and parents provide discrepant reports of child emotional well-being. Conceptual and clinical implications of these results are discussed.

## 1. Introduction

Incorporating reports of multiple informants who have varying perspectives is a recommended approach in the clinical assessment of children [1–6]. Complicating this process, reports of multiple informants regarding child emotional and behavioural functioning typically demonstrate low to moderate correlations, a finding that has been consistently documented across a variety of samples and measures (e.g., [3, 6–11]), with correspondence particularly low for child internalizing difficulties such as depression [5, 12]. Despite low correspondence, information from both parents and children can provide valuable insight into a child's functioning [4, 13] and each report may make unique contributions for making clinical judgments [14]. Thus, clinicians are often placed in the difficult position of deciding how to weigh these disparate reports in a case formulation or intervention for a given client [13, 15]. It is the aim of the present paper to examine parenting behaviours for improving prediction of early adolescent mood and emotional resilience beyond parent report of child emotional functioning. 

It is not surprising that parents and children often provide disparate reports of emotional functioning, given they have access to different information and experiences [13], hold different perspectives [11, 14], and may make different interpretations of the child's functioning and behaviour [4]. Additionally, parents' awareness of their child's inner thoughts and feelings strongly relies on parental inferences or what the child is willing to share [7], especially regarding less observable internalizing symptoms (e.g., sadness, loss of pleasure), which research consistently demonstrates are more difficult for parents to accurately recognize and report compared to the more observable and unambiguous externalizing symptoms [5, 8, 12, 14]. By definition, internal beliefs, feelings, and symptoms are also comparatively less likely to be directly observed by a clinician, and thus decisions regarding their presence may rely most heavily on reports of informants who know the child well, frequently the parent. 

Assessment of emotional resilience, reflecting concepts such as inner strength, competence, optimism, flexibility, and the ability to cope effectively when faced with adversity [16], is becoming increasingly common in clinical practice as resilient individuals are evidenced to be at decreased risk of developing psychopathology [17, 18] and promoting identification and building of child emotional resilience is increasingly emphasized in interventions aimed at decreasing depression, anxiety, and other youth psychopathology and increasing positive functioning (e.g., fun friends; [19]). Though emotional resilience has yet to be considered in report-discrepancy research, given the “internal” nature of the concept, it is likely that parents and children would demonstrate similar levels of concordance regarding youth emotional resilience as evidenced with reports of internalizing symptomology. Thus, understanding how to weigh parent and child reports of emotional resilience is likely to become of increasing importance in clinical practice. 

Given the fairly low correspondence between parents and children regarding children's internal experience, research is needed to identify specific factors that may be of use for informing how information provided by multiple informants on child mood and emotional functioning might best be interpreted [2] and incorporated to assess, diagnose, and provide treatment for at-risk children and youth most effectively. Extensive research has explored factors related to report discrepancy, and though research on family-related variables is beginning to increase, relatively, it is in its infancy [2, 20]. Research suggests that factors such as family conflict [15], family stress [5, 21], parental dysfunction [5], family communication, parental acceptance [20], and parental psychopathology [22] are related to report discrepancy. As such, understanding family context factors may be of particular value for informing interpretation of discrepant parent-child reports of child mood and emotional resilience when they inevitably arise. Understanding the family context in which disparate parent-child reports occur may be more important than attempting to reconcile disparate reports, as confronting informants regarding discrepancies may stress concordance versus accuracy [2]. Additionally, family context may be of importance during both assessment and intervention for child mood problems. In the present study, we consider two developmentally important parenting variables: parental responsiveness and psychological control and examine their ability to increase the prediction of child mood and emotional resilience beyond parent report of emotional functioning. 

Parental responsiveness encompasses expressing warmth and acceptance toward the child and devoting attention to the child's needs [23]. Lower levels of parent responsiveness may lead children to limit communication with parents to minimize conflict or exaggerate symptoms to elicit a response [24]. Indeed, unresponsive parents are generally less perceptive of their child's problems [25]. As a result, reports of their child's emotional well-being may reflect insensitivity to the youth's feelings and problems or be distorted from a lack of open parent-child communication. In contrast, in families with high parent responsiveness, children learn that they can trust their parents to respond to their problems with warmth and acceptance, which may lead to more accurate child disclosures and parent perceptions [5, 20]. 

Psychological control is a parenting behaviour in which parents attempt to manipulate children to adhere to parental standards through negative tactics such as guilt induction, shaming, isolation of the child, and love withdrawal [26]. Parents high in psychological control tend to be intrusive, demanding, hostile, emotionally manipulative and constrain child communication [27]. This acts to undermine the child's developing autonomy and sense of self [27] and keeps children emotionally dependent on parents [28]. Unlike behavioural control, reflecting supervision and monitoring [28], psychological control is an attempt to maintain power over a child and is indicative of a negative parent-child relationship [27, 28]. The combination of harsh judgment and focus on parental expectations rather than youths' needs may lead parents high in psychological control to be less attuned to their child's feelings and excessively harsh in their judgments and reports of their children's emotional functioning.

Both parental responsiveness and psychological control likely influence parent-child communication and parent ability to accurately understand, acknowledge, and report child emotional well-being. As such, knowledge of these parenting variables may be highly informative for clinicians when parent and child reports of mood and emotional functioning misalign. Given the pervasive nature of parent-child report discrepancies, the primary purpose of the present paper is to examine whether the consideration of parental responsiveness and psychological control improves ability to predict child mood and emotional resilience beyond parent reports of child emotional functioning. We specifically consider the child's perspective of parenting behaviour as the child's perception is more germane to parent-child report discrepancy in previous research (e.g., [20]). While previous research has considered parent-child report discrepancies in relation to responsiveness and acceptance-related parenting behaviours, to our knowledge, no previous research has considered the effect of psychological control, despite its relevance to child emotional functioning and hypothesized link to child disclosure and parent perception of child emotional functioning. Also, this is the first study to examine relatedness of parent and child perception of child emotional resilience and how parenting style may improve predication of this positive emotional construct. 

### 1.1. Hypotheses

We hypothesize that parent-child reports of both mood and emotional resilience will be moderately correlated. We also hypothesize that parental psychological control and responsiveness will significantly predict youth mood and emotional resilience after controlling for parent reports of each of these variables. Further, as previous research suggests parenting behaviours may result in varying effects on report discrepancy for mothers and fathers [20], we explore the effects of parenting variables for mothers and fathers individually.

## 2. Method

### 2.1. Participants

Participants were youth and their parents recruited from elementary schools in southwestern Ontario to participate in a longitudinal study on psychopathology in early adolescence. All students in four participating elementary schools in grades 5 to 8 (ages 9 to 15) were asked to bring home an information and consent package for parents to review. Of the 965 parents sent information packages, only 319 youth participated in the larger study. Fifty-one participants were excluded due to missing more than 25% of data on measures of interest. This left a final sample of 268 children (132 boys and 136 girls) and their parents (36 fathers, 154 mothers, 78 not reported). Children ranged in age from 9 to 15 (*M* = 11.69, SD = 1.08). Consistent with the demographics in this community, the participants were mainly White (*n* = 219), but also included Asian or Asian Canadian (*n* = 24), Black or African Canadian (*n* = 2), Hispanic or Latino (*n* = 6), First Nations (*n* = 1), and other (*n* = 16) ethnicity. Most participants (*n* = 153) did not complete information on family structure, yielding a variable that was not particularly informative. Of those who did report family structure (*n* = 115), 85 reported parents who were married or living together, 17 reported parents were separated, 12 reported divorced parents, and 1 reported parents were remarried. 

From the larger sample, a subsample of 92 pairs of parent (14 fathers, 71 mothers, 7 not reported) and child (44 boys and 48 girls) participants provided reports of child resilience. (Sample sizes are not equal for mood and emotional resilience analyses as measures of emotional resilience were added to the larger study at a later stage of the project.) These participants did not differ from the participants who did not complete resilience measures, in regards to age *t*(144.99) = 1.47, *ns, *sex *t*(266) = .34, *ns,* ethnicity *t*(263) = −.75, *ns*, CDI score *t*(266) = −.32, *ns*, father responsiveness *t*(266) = 1.28, *ns*, father psychological control *t*(266) = .99, *ns*, mother responsiveness *t*(266) = 1.86, *ns*, mother psychological control *t*(266) = −.27, *ns*, total parental responsiveness *t*(266) = 1.80, total parental control *t*(266) = .39, *ns,* parental rating of positive affect *t*(266) = 1.38, *ns*, or parental rating of negative affect *t*(266) = 1.58, *ns*.

### 2.2. Measures

#### 2.2.1. Child Mood

To assess the child's self-reported mood, youth completed the Child Depression Inventory (CDI; [29]). The CDI is a self-report scale designed for children aged 7 to 17 to assess the number and severity of depression symptoms over the preceding two weeks, including mood disturbances, self-evaluation, feelings of pleasure, sleep disturbance, and interpersonal behaviours. Each item contains three statements scored on a three-point scale (0 = *absence of symptoms, *1 = *mild symptom*, 2 = *definite symptom*). The CDI evidences high internal consistency, test-retest reliability, and construct validity [30, 31]. As clinical followup was not readily available, the item assessing suicide ideation was omitted. The Cronbach's alpha was  .93 in the present sample.

#### 2.2.2. Parental Report of Child Mood

Parent report of child mood was assessed using the Positive and Negative Affect Scale-Parent Version (PANAS-P; [32]). (As parent reported CDI was unavailable, positive and negative affect from the Positive and Negative Affect Scale-Parent Version were used as proxies for capturing child mood.) The 30-item scale requires parents to rate mood-related adjectives as they relate to their child on a 5-point scale ranging from 1 (*very slightly or not at all) *to 5 (*extremely)*. The PANAS demonstrates adequate internal consistency, test-retest reliability, convergent validity, and predictive validity [33]. In the present sample, the Cronbach's alpha for the negative affect subscale was  .89 and  .91 for the positive affect subscale.

#### 2.2.3. Emotional Resilience

Parents and children reported child emotional resilience using the Resilience Scale [34]. This 25-item scale requires parents and children to rate items regarding how the child copes with everyday challenges on a 7-point scale ranging from “disagree completely” to “agree completely.” The Resilience Scale has been found to have adequate internal consistency, concurrent validity, convergent, and discriminant validity [35]. In the present sample, the Cronbach's alpha was  .95 for the parent reported child resilience and  .93 for child's self-reported resilience.

#### 2.2.4. Child Report of Parental Responsiveness and Psychological Control

To assess the child's perceptions of parenting behaviours, subscales from the shortened version of the Child Report of Parent Behaviour Inventory (CRPBI; [36]) related to responsiveness (e.g., “My mother makes me feel better after talking over my worries with her”) and psychological control (e.g., “My father brings up past mistakes when he criticizes me”) were administered. Children indicated the extent to which they agreed with 14 statements (6 items responsiveness, 8 items psychological control) separately for both mother and father on a scale ranging from 1 (*disagree*) to 5 (*agree*). The CRPBI has demonstrated good reliability, internal consistency [37], convergent, and discriminant validity [38]. In the present study the Cronbach's alphas for the responsiveness subscale were  .87 for ratings of mothers and  .92 for ratings of fathers. For the psychological control subscales, the Cronbach's alphas were  .77 for ratings of mothers and  .81 for ratings of fathers.

### 2.3. Procedure

Undergraduate research assistants visited each participating classroom to invite youth from grade 5 to 8 to participate in a larger study from which the current study is based. Consent forms were sent home with each potential participant along with parent questionnaires to be completed at home and returned to the school prior to the in-school data collection. 

For the data collection, research assistants brought groups of participants to a separate room within the school that had been set up as a mobile computer lab consisting of approximately 25 10′ Asus Netbooks, each with a privacy shield. Prior to completing the measures, children were informed that participation was voluntary and confidential and gave their assent to participate. All measures were completed on the computer in approximately one hour. Children were encouraged to ask questions, if needed, while completing the tasks. Children completed the Resilience Scale prior to the Child Report of Parenting Behaviours Inventory and then the Child Depression Inventory among other study measures. (These data were part of a larger data set including measures of cognitive schemas, anxiety, and self-esteem in addition to current study measures.) Research assistants circulated the room to provide aid as necessary.

### 2.4. Statistical Methods

Data analysis was carried out according to the following steps. First, for participants missing less than 25% of data, scores were prorated using the average of the remaining items. Next, we examined the effects of several demographic variables in relation to the measures of interest used in the study. We then examined the correlations between child and parent reports of child mood and emotional resilience. Finally, for the primary analyses, we conducted several hierarchical multiple regression analyses with child-reported mood or emotional resilience as the criterion. Hierarchical regression was an appropriate approach given our interest in examining how parenting behaviours might predict variance in child mood and emotional resilience beyond that accounted for by parent reports of those constructs. Demographic variables found to be related to other study measures were entered into the first regression block of each equation, and parent report of child mood or emotional resilience were entered in the second block. Finally, to determine whether parenting behaviours improve ability to predict child-reported mood and emotional resilience beyond reports of parents, these variables were entered into the final block of each equation.

## 3. Results

### 3.1. Descriptive Characteristics of Sample

Descriptive statistics are presented in Table 1 for the entire sample and then for each sex and ethnicity. We first examined the effects of sex, age, ethnicity, and family status in relation to the measures used in the study. Age and family structure were not significantly related to any measures of interest (all *P*′*s* > .05). Child's sex was related to father psychological control *t*(256.23) = −2.24, *P* = .03, with boys reporting higher levels than girls. Ethnicity was also related to father psychological control, *t*(266) = −2.41, *P* = .02, mother psychological control, *t*(58.73) = −2.10, *P* = .04, and parental psychological control, *t*(59.50) = −2.31, *P* = .02, with youth reporting Caucasian ethnicity also reporting lower levels of control than those reporting diverse ethnicities on all three measures of psychological control. Therefore, the effects of child sex and ethnicity will be controlled for in all subsequent analyses.

### 3.2. Child and Parent Perceptions of Child Emotional Functioning

Correlations for all study variables are available upon request. Consistent with hypotheses, child-reported depression scores were moderately correlated with parent-reported child negative affect (*r* = .20, *P* = .001) and moderately negatively correlated with parent reported child positive affect (*r* = −.27, *P* < .001). Child-reported emotional resilience scores were also moderately correlated with parent-reported child emotional resilience (*r* = .28, *P* = .01).

### 3.3. Improving Prediction of Child Mood beyond Parent Report

The central goal of the present research was to examine how parenting variables may contribute to the prediction of child mood and emotional resilience beyond parent reports of these constructs. A hierarchical linear regression was performed using the child's rating of mood as the dependent variable. Given preliminary analyses suggesting that child sex and ethnicity were significantly associated with parenting variables, these variables were entered into the first block as controls, and parent report of positive and negative affect was entered into the second block of the equation. Finally, ratings of parental responsiveness and psychological control were entered into the regression equation in the final block. 

Results are summarized in Table 2. Together, parental responsiveness and psychological control were found to significantly predict child depression scores after controlling for the parent ratings of affect, child sex, and ethnicity, *F*(6,261) = 18.60, *P* < .001. Parenting behaviors explained a significant 20% increase in proportion of variance in child depression, *F*(2,261) = 36.52, *P* < .001. In addition, parental responsiveness and parental psychological control were each found to make significant contributions in predicting child depression scores after controlling for all other variables in the analysis.

### 3.4. Improving Prediction of Child Emotional Resilience beyond Parent Report

To examine how parenting behaviors might help predict child emotional resilience beyond parent report of child emotional resilience, a second hierarchical linear regression was performed with child's self-reported emotional resilience score as the dependent variable. Child sex and ethnicity were entered into the first block of the regression equation as controls, and parent-reported child emotional resilience was entered into the second block of the equation. Finally, ratings of parental responsiveness and psychological control were entered into the regression equation in the final block. 

Results are summarized in Table 3. Together, parental responsiveness and psychological control significantly predicted child's emotional resilience scores after controlling for parent rating of child emotional resilience, child sex, and ethnicity *F*(5,86) = 8.81, *P* < .001. Again, parenting behaviours explained a significant 25% increase in proportion of variance in the child emotional resilience scores *F*(2,86) = 16.14, *P* < .001. In this analysis, only parental responsiveness (and not psychological control) emerged to significantly predict child emotional resilience after controlling for the other variables.

### 3.5. Mother versus Father Parenting Behaviours in Predicting Child Emotional Functioning

To more fully examine the role of the perception of mother and father behaviour separately, a more fine-grained analysis was undertaken. In this analysis we explored mother and father responsiveness and psychological control variables individually. 

#### 3.5.1. Prediction of Child Mood

To examine how the behaviour of each parent may uniquely contribute to the prediction of child mood beyond a parent's report of child mood, a hierarchical linear regression was performed using the child's self-reported mood as the dependent variable. Again, child sex and ethnicity were entered into the first block of the equation as controls, and parent-reported child positive and negative affect were entered into the second block. Mother and father responsiveness and mother and father psychological control were entered into the final block of the equation. 

Results are summarized in Table 4. Mother responsiveness, *t*(261) = −2.02, *P* = .04, mother control, *t*(261) = 2.81, *P* = .005 and father responsiveness, *t*(261) = −2.67, *P* = .008, each significantly predicted child depression scores beyond the parent rating of child mood.

#### 3.5.2. Prediction of Child Emotional Resilience

To examine how the behavior of each parent may uniquely contribute to the prediction of child emotional resilience beyond a parent's report, a hierarchical linear regression was performed using the child's self-reported emotional resilience as the dependent variable. Again, child sex and ethnicity were entered into the first block of the equation as controls, and parent-reported child emotional resilience was entered into the second block. Mother and father responsiveness and mother and father psychological control were entered into the final block of the equation. 

Results are summarized in Table 5. Mother responsiveness, *t*(86) = 2.73, *P* = .008 and father responsiveness, *t*(86) = 2.56, *P* = .01, each significantly predicted child emotional resilience beyond the parent rating of child emotional resilience.

## 4. Discussion

The central objective of this study was to examine how parental responsiveness and psychological control contribute to the prediction of child mood and emotional resilience beyond parent reports of these constructs. First, consistent with hypotheses, parent and child reports of both mood and emotional resilience were only moderately correlated in this early adolescent sample. This is consistent with previous research suggesting that parents may struggle with fully recognizing internalizing symptoms in their children [5, 8, 12, 14]. Parents may be reluctant to notice or acknowledge troubling mood symptoms in their child at least in part due to the distress this knowledge may elicit in the parent. Yet, for the first time, we also demonstrate that parent and child reports of child emotional resilience, a positive emotional construct, are also moderately related, suggesting parents may lack concordance when it comes to awareness of a child's positive emotional functioning as well.

Second, and also consistent with hypotheses, total parental responsiveness and psychological control were together found to significantly predict child depression symptoms beyond parent's rating of affect. Together, these parenting behaviours increased the variance accounted for in child-reported mood by 20%, after controlling for parent report, child sex, and ethnicity. More specifically, low levels of parental responsiveness and high levels of psychological control each predicted a significant portion of the variance in child mood after parent reports were accounted for. When considering child emotional resilience, though together, parental responsiveness and psychological control increased the variance accounted for by 25% in the model, only parental responsiveness was significantly predictive beyond parent report of child emotional resilience. Unexpectedly, parental psychological control did not add significant explanatory power to the emotional resilience model. Thus, parental psychological control appeared to be more specifically predictive of negative (child depression) versus positive (child emotional resilience) constructs. These results suggest that when it comes to understanding aspects of a child's emotional functioning not accounted for by parent report, a warm and accepting parental relationship may be important in the development of both depression and emotional resilience, while in a multivariate context, psychological control appears to be more specifically linked to the development of depression. 

Results regarding the explanatory power of parental responsiveness in these models are somewhat consistent with previous research on report discrepancy and parent-child attachment [24] and child acceptance [5, 20]. However, this is the first study to our knowledge that identifies parental responsiveness for contributing to the prediction of child emotional resilience beyond parent reports. This study is also the first to suggest that psychological control explains variance in child depression not accounted for by parent reports of child mood. This finding is not surprising in light of the strong association between the use of psychological control and negative developmental outcomes (e.g., [28, 39]). This finding is also consistent with developmental models of depression vulnerability stressing the importance of highly critical parenting in laying the foundation for cognitive risk for depression (e.g., [40]). Despite its importance in our model predicting child depression, psychological control has been neglected in the parent-child report discrepancy literature. Our research suggests this construct bears further consideration in theoretical models attempting to illuminate parent reports of child mood and also that knowledge of parent psychological control may be of value for clinicians assessing parent reports of child depression.

Research on parenting behaviours, child mood, and emotional functioning tends to focus almost exclusively on the mother. To further explore whether the importance of parenting behaviour in these models is dependent on *which parent* is perceived to exhibit the behaviour, we examined the same models considering mother and father responsiveness and psychological control separately. For the mood analysis, father and mother responsiveness and mother's use of psychological control were each found to significantly predict child depression symptoms beyond parent ratings of affect. Interestingly, it was *maternal *psychological control that explained the largest portion of variance in child depression scores in this model. In many families, mothers continue to bear primary responsibility for childrearing and may be more likely typified as the warmer, more nurturing parent. This context may make maternal use of psychological control especially harmful to child development. 

For the emotional resilience analysis, father and mother responsiveness both significantly improved the ability to predict child emotional resilience beyond the parent-reported child emotional resilience and control variables. Neither mother or father psychological control emerged as significant predictors of child emotional resilience, again suggesting that in a multivariate context, this parenting construct is more specifically implicated when examining the relation between parent and child reports of mood. This research suggests that responsiveness may be predictive more generally to positive outcomes beyond parental reports, while psychological control may be a better, more specific predictor of child mood versus emotional resilience. Additionally, the present study supports *both* mother and father parenting behaviours as making unique contributions to child emotional functioning beyond parent reports, underscoring the importance of including both parents (when circumstances allow) in research and clinical decision making, rather than relying heavily on mothers alone. 

This study highlights the importance of considering family relationships, including specific parenting behaviours, when addressing disparate reports of youth emotional functioning. Parenting behaviours, such as responsiveness and psychological control, may provide information about parents' level of awareness of the child's mood. As highlighted by the present research, disparate reports may also provide valuable insight regarding the family relationship. Rather than struggling to determine which informant's report is more accurate or valuable, discrepancies may be considered as an indication to further explore the relationship between informants to determine why the discrepancy has occurred. Therapeutically, examining disparate reports of child emotional functioning with children and their parents may also provide an opportunity to address family perceptions and expectations, as well as to improve family communication and the family's ability to recognize and cope with a child's mood difficulties. 

Limitations of the present study require note. First, child mood was reported as depression symptoms by children and as positive and negative affect by parents. While considerable research has documented that depression symptoms are related to high levels of negative affect and low levels of positive affect [41, 42], affect measures are theorized to measure relatively stable traits, whereas depression symptoms are more likely to fluctuate over time. The use of parallel measures would have been ideal, though some researchers have argued for using related but not parallel measures in other report discrepancy researches (e.g., [10, 43]) and some have even suggested that parallel measures may be no more ideal [44]. Additionally, much report discrepancy research focuses around difference scores, making parallel measures more essential. This study utilizes a regression approach, somewhat ameliorating this concern. Furthermore, at least one study has shown that using parallel measures does not improve concordance between mother and child reports of internalizing symptoms [43]. Finally, related, rather than parallel measures are often used in clinical evaluation of youth [43].

Although we suggest that parenting behaviours may influence a parent's ability to accurately intuit a child's mood and emotional resilience, it might also be that when youth are feeling depressed they also tend to have a negatively biased view of parenting behaviour. Although possible, considerable previous research suggests that youth do quite accurately report parenting experiences, independent of current level of depression, especially when specific parenting behaviours are examined as they were in the current study (e.g., [45]). Furthermore, as earlier stated we are most interested in the child's *perception* of the parent versus actual parent behaviour, and this could be influenced by a host of factors including child mood. 

Given that child perceptions of parenting may be more closely related to parent-child report discrepancies than parent perceptions of their own behaviour (e.g., [20]), considering the child's perspective of parenting is a strength of the present research design. Nevertheless, a multimethod assessment of parenting behaviours incorporating observational measures would be valuable and allow comparison of the role of child perception and actual parenting behaviours in accounting for disparate reports of child emotional functioning. 

 To maximize participation, we were unable to ensure equal participation from mothers and fathers. All children in the study do report on both mother and father behaviour, and given that we were most interested in examining the child's perception of parenting behaviours, we included all participants meeting study criteria to maximize our sample size.

Participants in the present study were youth volunteers from a community versus a clinical setting, and we must await findings from clinical research to know more certainly how these patterns would be present in a clinic-referred sample of youth. Sex differences may also be important to further explore as in the present study boys reported higher levels of father psychological control than girls. It is possible that boys perceive higher levels of control than girls, or it may be that fathers indeed exert more psychological control over sons than daughters. Regardless of the reason for this difference, child sex was controlled in all analyses, limiting the impact on our results. Though difficult to draw conclusions given the limited diversity of the present sample, we did find ethnic differences in youth's perception of mother, father and total psychological control, with youth of Caucasian ethnicity reporting lower levels than diverse ethnicities. Indeed, much research suggests that there are cultural differences in the level of control exerted by parents (e.g., [46]). While some research suggests that psychological control is related to negative outcomes across a wide variety of cultures (e.g., [47]), other researches demonstrate that psychological control may relate to specific outcomes differently across cultures (e.g., [46, 48]). Additional research is necessary to determine how the use of psychological control may influence parents' understanding of their child's emotional functioning differently across cultures. 

In conclusion, the use of multiple informants for assessing youth mood and emotional functioning is typical in clinical practice. Rather than focusing on discrepancies between these reports as a problem, increasing understanding of family contextual factors that underlie parent-child inconsistency about a child's mood and emotional resilience may present an important opportunity to improve the quality and outcome of treatment, rather than hinder it.

## Figures and Tables

**Table 1:** Means and standard deviations for parent and child ratings of mood and emotional resilience and child rating of parenting behaviours by sex, ethnicity and for total sample.

	Total		Male		Female		Caucasian		Diverse	
	*n* = 268		*n* = 132		*n* = 136		*n* = 219		*n* = 49	
	*M*	SD	*M*	SD	*M*	SD	*M*	SD	*M*	SD
*Child Rating *										
Depression symptoms	7.43	7.16	8.07	8.10	6.81	6.07	7.13	6.54	8.75	9.41
Mother responsiveness	4.40	.73	4.41	.73	4.39	.73	4.43	.66	4.25	.98
Mother control	1.83	.78	1.87	.82	1.78	.75	1.77*	.71	2.09*	1.02
Father responsiveness	4.05	.90	4.08	.90	4.03	.90	4.05	.91	4.09	.84
Father control	1.92	.75	2.02*	.81	1.82*	.68	1.87*	.71	2.15*	.88
Total parental responsiveness	8.45	1.40	8.48	1.42	8.42	1.38	8.48	1.37	8.33	1.51
Total parental control	3.75	1.37	3.90	1.46	3.60	1.27	3.64*	1.25	4.24*	1.74
Resilience	134.71	22.44	134.77	22.90	134.65	22.25	130.92	21.67	131.47	22.64
*Parent Rating*										
Child positive affect	53.91	8.73	53.08	9.07	54.72	8.34	53.64	8.69	55.12	8.88
Child negative affect	23.66	7.25	24.34	7.82	23.01	6.62	23.61	7.38	23.92	6.72
Resilience	127.28	22.77	125.84	21.87	128.60	23.71	127.42	22.25	126.71	24.37

*P < .05

**Table 2:** Effect of parental responsiveness and psychological control in predicting child mood after controlling sex, ethnicity, and parent-reported child positive and negative affect (*N* = 268).

Variable	*B*	SEB	*β*	*R*²	Δ*R* ^2^
Step 1				.01	
Sex	1.15	.88	.08		
Ethnicity	1.48	1.13	.08		
Step 2				.10***	.09***
Parent reported PA	−.19	.05	−.24***		
Parent reported NA	.14	.06	.14*		
Step 3				.30***	.20***
Responsiveness	−1.36	.31	−.27***		
Control	1.48	.31	.28***		

Analysis includes 36 fathers, 154 mothers and 78 not reported

*P < .05

**P < .01

***P < .001

**Table 3:** Effect of parental responsiveness and psychological control in predicting child emotional resilience after controlling sex, ethnicity, and parent-reported child emotional resilience (*N* = 92).

Variable	*B*	SEB	*β*	*R* ^2^	Δ*R* ^2^
Step 1				.01	
Sex	−.97	4.85	−.02		
Ethnicity	5.70	5.87	.11		
Step 2				.09*	.08**
Parent reported resilience	.28	.10	.28**		
Step 3				.34***	.25***
Responsiveness	8.35	1.49	.52***		
Control	1.25	1.54	.08		

Analysis includes 14 fathers, 71 mothers and 7 not reported

*P < .05

**P < .01

***P < .001

**Table 4:** Effect of mother and father responsiveness and psychological control in predicting child mood after controlling sex, ethnicity, and parent-reported child positive and negative affect (*N* = 268).

Variable	*B*	SEB	*β*	*R*²	Δ*R*²
Step 1				.01	
Sex	1.15	.88	.08		
Ethnicity	1.48	1.13	.08		
Step 2				.10***	.09***
Parent reported PA	−.19	.05	−.24***		
Parent reported NA	.14	.06	.14*		
Step 3				.30***	.20***
Mother responsiveness	−1.37	.68	−.14*		
Mother control	1.87	.67	.21**		
Father responsiveness	−1.35	.51	−.17**		
Father control	1.06	.67	.11		

Analysis includes 36 fathers, 154 mothers and 78 not reported

*P < .05

**P < .01

***P < .001

**Table 5:** Mother and father responsiveness and psychological control in predicting child emotional resilience after controlling sex, ethnicity, and parent-reported child emotional resilience (*N* = 92).

Variable	*B*	SEB	*β*	*R*²	Δ*R*²
Step 1				.01	
Sex	−.97	4.85	−.02		
Ethnicity	5.70	5.87	.11		
Step 2				.09*	.08**
Parent reported resilience	.28	.10	.28**		
Step 3				.35***	.26***
Mother responsiveness	10.72	3.93	.31**		
Mother control	−.79	4.01	−.03		
Father responsiveness	7.07	2.76	.29**		
Father control	3.35	3.84	.12		

Analysis includes 14 fathers, 71 mothers and 7 not reported

*P < .05

**P < .01

***P < .001
